# Complex Positive Effects of SGLT-2 Inhibitor Empagliflozin in the Liver, Kidney and Adipose Tissue of Hereditary Hypertriglyceridemic Rats: Possible Contribution of Attenuation of Cell Senescence and Oxidative Stress

**DOI:** 10.3390/ijms221910606

**Published:** 2021-09-30

**Authors:** Jaroslava Trnovska, Petr Svoboda, Helena Pelantova, Marek Kuzma, Helena Kratochvilova, Barbora Judita Kasperova, Iveta Dvorakova, Katerina Rosolova, Hana Malinska, Martina Huttl, Irena Markova, Olena Oliyarnyk, Magdalena Melcova, Vojtech Skop, Milos Mraz, Sona Stemberkova-Hubackova, Martin Haluzik

**Affiliations:** 1 Cardiometabolic Research Division, Centre for Experimental Medicine, Institute for Clinical and Experimental Medicine, 140 21 Prague, Czech Republic; svobodae@vscht.cz (P.S.); helena.kratochvilova@ikem.cz (H.K.); iveta.dvorakova@ikem.cz (I.D.); hana.malinska@ikem.cz (H.M.); martina.huttl@ikem.cz (M.H.); irena.markova@ikem.cz (I.M.); ooliyarnyk@yahoo.com (O.O.); vojtech.skop@nih.gov (V.S.); 2 Laboratory of Animal Biochemistry, Department of Biochemistry and Microbiology, University of Chemistry and Technology Prague, 166 28 Prague, Czech Republic; katerina.rosolova@ikem.cz (K.R.); melcovam@vscht.cz (M.M.); 3 Laboratory of Molecular Structure Characterization, Institute of Microbiology, Czech Academy of Sciences, 142 20 Prague, Czech Republic; pelantova@biomed.cas.cz (H.P.); kuzma@biomed.cas.cz (M.K.); 4 Department of Analytical Chemistry, Faculty of Science, Palacky University Olomouc, 779 00 Olomouc, Czech Republic; 5 Diabetes Centre, Institute for Clinical and Experimental Medicine, 140 21 Prague, Czech Republic; barbora.judita.kasperova@ikem.cz (B.J.K.); milos.mraz@ikem.cz (M.M.); 6 Laboratory of Molecular Therapy, Institute of Biotechnology, Czech Academy of Sciences, 252 50 Prague, Czech Republic; stemberkova@ibt.cas.cz; 7 Institute of Medical Biochemistry and Laboratory Diagnostics, First Faculty of Medicine, Charles University and General University Hospital, 128 08 Prague, Czech Republic

**Keywords:** empagliflozin, metabolic syndrome, hypertriglyceridemia, insulin sensitivity, cell senescence, hereditary hypertriglyceridemic rat model

## Abstract

(1) Background: empagliflozin, sodium-glucose co-transporter 2 (SGLT-2) inhibitor, is an effective antidiabetic agent with strong cardio- and nephroprotective properties. The mechanisms behind its cardio- and nephroprotection are still not fully clarified. (2) Methods: we used male hereditary hypertriglyceridemic (hHTG) rats, a non-obese model of dyslipidaemia, insulin resistance, and endothelial dysfunction fed standard diet with or without empagliflozin for six weeks to explore the molecular mechanisms of empagliflozin effects. Nuclear magnetic resonance (NMR)-based metabolomics; quantitative PCR of relevant genes involved in lipid and glucose metabolism, or senescence; glucose and palmitic acid oxidation in isolated tissues and cell lines of adipocytes and hepatocytes were used. (3) Results: empagliflozin inhibited weight gain and decreased adipose tissue weight, fasting blood glucose, and triglycerides and increased HDL-cholesterol. It also improved insulin sensitivity in white fat. NMR spectroscopy identified higher plasma concentrations of ketone bodies, ketogenic amino acid leucine and decreased levels of pyruvate and alanine. In the liver, adipose tissue and kidney, empagliflozin up-regulated expression of genes involved in gluconeogenesis and down-regulated expression of genes involved in lipogenesis along with reduction of markers of inflammation, oxidative stress and cell senescence. (4) Conclusion: multiple positive effects of empagliflozin, including reduced cell senescence and oxidative stress, could contribute to its long-term cardio- and nephroprotective actions.

## 1. Introduction

The sedentary lifestyle accompanied by excessive energy intake leads to metabolic imbalance and increased risk of type 2 diabetes mellitus (T2DM), dyslipidaemia, arterial hypertension, non-alcoholic fatty liver disease (NAFDL) and other pathologies commonly referred to as metabolic or insulin resistance syndrome [1]. A combination of these diseases, in turn, contributes to increased cardiovascular morbidity and mortality. Obese patients with T2DM typically suffer from subclinical inflammation that is involved in tissue damage and premature cell aging. An increased amount of senescent cells in different tissues and organs has been found in experimental models of T2DM and obesity [2]. Importantly, senescent cells could participate in the onset and progression of multiple pathologies present in patients with T2DM. Moreover, their pro-inflammatory secretory phenotype supports the spreading of senescence, which contributes to the further progression of the related diseases [2,3,4].

The SGLT-2 inhibitor empagliflozin is widely used for the treatment of T2DM, and it has been shown to be effective in reducing cardiovascular complications and related pathologies. EMPA-REG OUTCOME trial [5], EMMY trial [6] or EMPEROR-Reduced trial [7] documented significant benefits of empagliflozin in the reduction of cardiovascular mortality, hospitalization for heart failure and death from any cause along with nephroprotection [5,6,7].

Numerous hypotheses have been put forward to explain the positive effects of empagliflozin and other drugs from the SGLT-2 inhibitor class on cardiovascular and renal outcomes. One of the hypotheses suggested that empagliflozin may optimise cardiac and renal energy metabolism by a shift from lipid and glucose oxidation towards ketone bodies. Higher levels of the main ketone β-hydroxybutyrate in circulation could then serve as a fuel for myocardial cells and increase their mechanical efficiency [8,9,10,11]. Empagliflozin is also effective in body weight reduction and blood pressure lowering [5] and on reducing markers of inflammation [12].

We hypothesised that, in addition to known mechanisms of action, inhibition of SGLT-2, empagliflozin could also act through modification of the number of senescent cells in different tissues and organs. This work was undertaken to explore the molecular mechanisms of empagliflozin effects related to improved metabolic status and the amelioration of renal and cardiovascular complications. To this end, we used a combination of nuclear magnetic resonance (NMR)-based metabolomics and gene expression profiling in adipose tissue, liver and kidney in the hereditary hypertriglyceridemic rats (hHTG) that represent a lean non-diabetic model of insulin resistance, dyslipidaemia and related complications.

## 2. Results

### 2.1. Empagliflozin Had Beneficial Effect on Body Weight, Glycaemia and Lipidaemia

**Figure 1:**
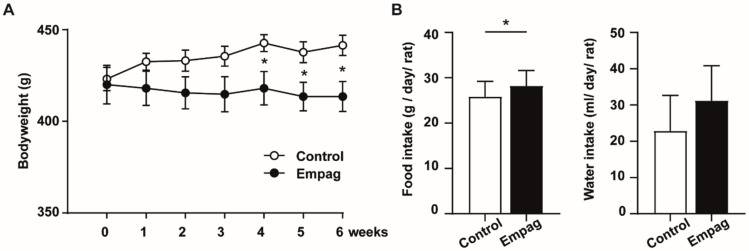
Body weight, food and water consumption. (**A**) Body weight during 6 weeks of treatment of hHTG rats by empaglifozin compared to control hHTG rats. (**B**) Daily food and water intake are expressed as a food/water intake by cage and calculated by averaging for one animal/day over the entire course of the study. Control group (*n* = 7) and empagliflozin group (*n* = 8). The empagliflozin group received a standard diet enriched by 0.01% empagliflozin for 6 weeks. Data are expressed as mean ± SEM; * *p* ˂ 0.05.

Empagliflozin treatment for six weeks attenuated body weight gain (Figure 1A) despite food intake, but not water intake was greater in empagliflozin treated rats (Figure 1B).

Increased glycosuria was detected after empagliflozin administration, while almost no glucose was detected in the urine of the control group (Table 1). Empagliflozin administration decreased fasting glycaemia. However, no significant difference was observed in postprandial glucose concentrations (Table 1). Plasma insulin decreased in empagliflozin compared to the control group (Table 1). Non-fasting plasma triglycerides (TAG) were decreased by empagliflozin treatment along with HDL-cholesterol increase, while total cholesterol did not significantly change. The Non-esterified fatty acids (NEFA) level in plasma was increased in empagliflozin compared to the control group. Plasma leptin levels were also reduced (Table 1).

### 2.2. Empagliflozin Reduced Oxidative Stress in the Liver and Modified Genes Involved in Lipid Metabolism Pathway 

**Figure 2:**
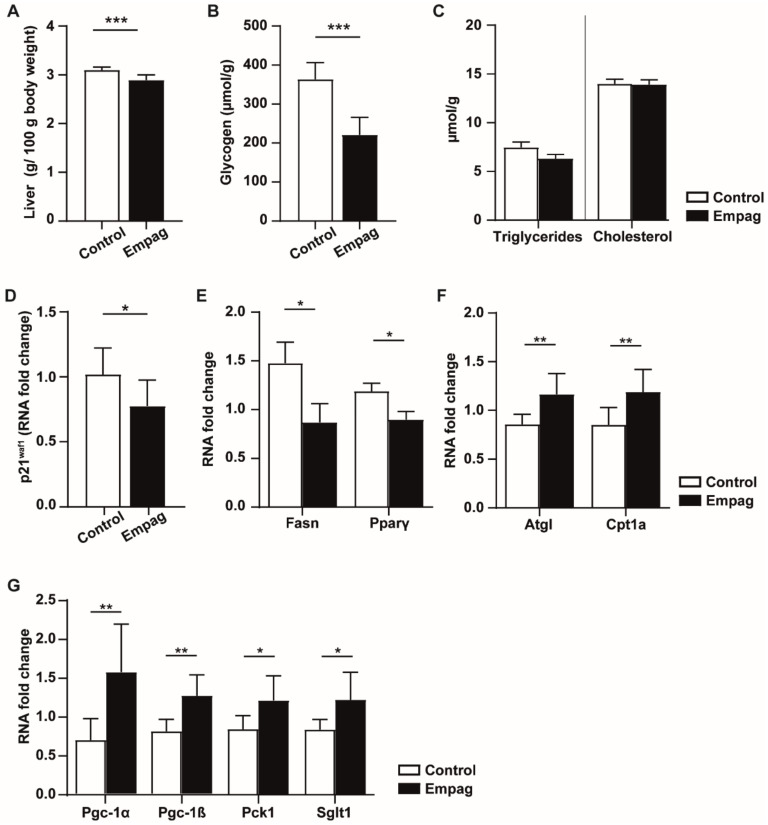
Effect of empagliflozin on the liver. (**A**) Relative weight of the liver is expressed as the weight of the liver related to 100 g of body weight. (**B**) Glycogen concentration in the liver measured by the Anthron method. (**C**) Triglycerides and total cholesterol concentrations are related to the weight of tissue. (**D**–**G**) Changes of mRNA level of selected genes involved in senescence and lipid metabolism pathway. Control group (*n* = 7) and empagliflozin group (*n* = 8). Empagliflozin group received a standard diet enriched by 0.01% empagliflozin for 6 weeks. Data are expressed as mean ± SEM; * *p* ˂ 0.05; ** *p* ˂ 0.01; *** *p* ˂ 0.001.

We next studied the effect of empagliflozin on the liver. Empagliflozin treatment significantly lowered relative liver weight compared to the control group (Figure 2A). Empagliflozin administration was associated with a 39% decrease in glycogen content (Figure 2B), but no effect on TAG and cholesterol levels was observed in the liver (Figure 2C).

Empagliflozin increased the activity of glutathione peroxidase (GSH-Px) and glutathione reductase (GR) (Table 2). The activity of catalase (CAT) was greater in empagliflozin group compared to untreated controls while thiobarbituric acid reactive substances (TBARS) were decreased (Table 2). These data indicate that empagliflozin increases antioxidant defence in the liver. Increased oxidative stress is a well-known inducer of premature senescence. There is evidence that senescent hepatocytes are a major driver of liver steatosis, possibly via the inability of mitochondria to efficiently metabolize fatty acids with subsequent excessive lipid accumulation in the liver [13].

We detected that empagliflozin treatment down-regulated liver expression of the senescence marker *p21^Waf1^* (Figure 2D) together with genes involved in lipid storage: *Fasn* and *Ppar*γ (Figure 2E). Moreover, empagliflozin up-regulated the expression of genes involved in lipid catabolism, such as adipose triglyceride lipase (*Atgl*) or carnitine palmitoyl transferase transporter (*Cpt1a*; Figure 2F). Furthermore, we observed increased expression of *Pgc-1α* and *Pgc-1β*, well-known regulators of mitochondrial biogenesis and up-regulation of *Pck1* involved in gluconeogenesis together with glucose transporter *Sglt1* suggesting induction of hepatic glucose production (Figure 2G).

To further explore the effect of empagliflozin on hepatocytes, we used the HepG2 hepatocytes cell line, which was used to study SGLT-2 inhibitors [14,15]. The viability of HepG2 cells treated with empagliflozin was measured. We tested empagliflozin at concentrations of 1, 10, 100 and 1000 nM in medium with 5 mM glucose concentration. Empagliflozin did not affect cellular viability (Figure S1A) and for subsequent experiments, Empagliflozin was used at 500 nM, which was comparable to our *in vivo* experiments.

**Figure 3:**
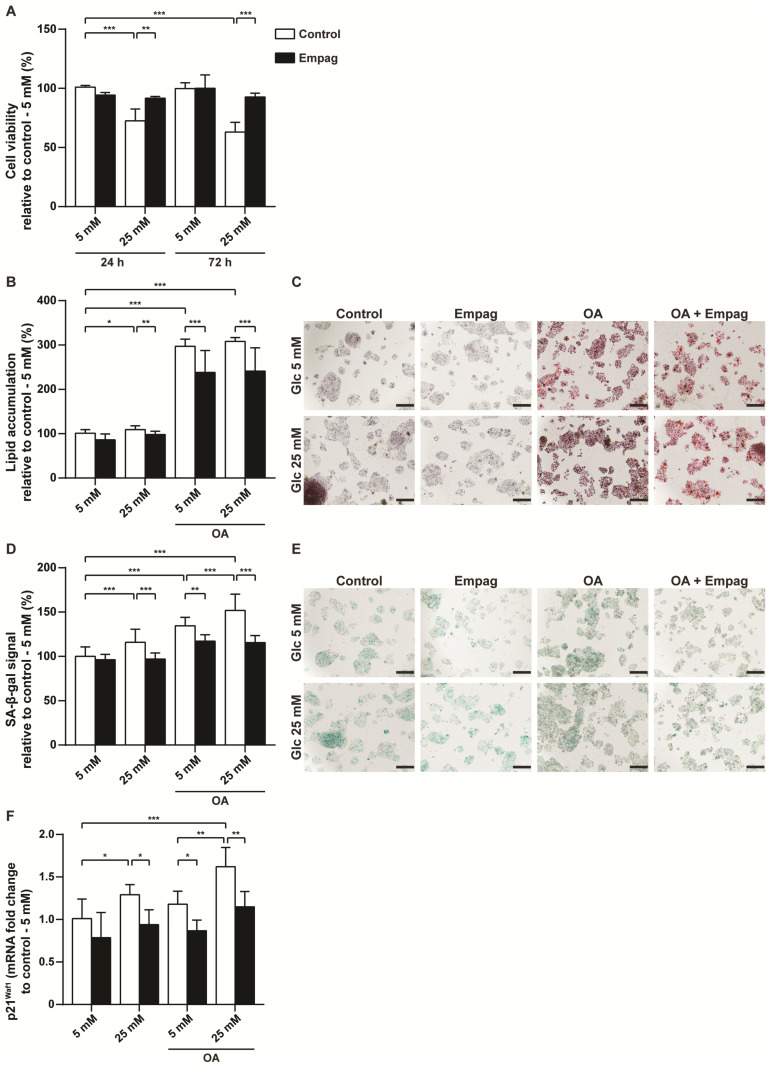
Effect of empagliflozin on HepG2 hepatocytes. HepG2 cells were exposed to glucose (5 mM or 25 mM), 1.5 mM oleic acid (OA) together with 500 nM empagliflozin treatment for 2 days. (**A**) Determination of cell viability assessed by WST-1 kit. (**B**) Quantification of intracellular lipid accumulation using Oil Red O staining was performed and (**C**) representative images are shown. The bar indicates 200 μm. (**D**) Detection of senescent cells evaluated on senescence-associated beta-galactosidase (SA-β-gal) activity and (**E**) representative images are shown. The bar indicates 200 μm. (**F**) Changes of mRNA level of senescence marker *p21^Waf1^*. The results are derived from at least three independent experiments run in triplicates. Data are expressed as mean ± SD; * *p* < 0.05; ** *p* < 0.01; *** *p* < 0.001.

HepG2 cells were treated with 500 nM empagliflozin and cultured in physiological Minimum Essential Medium (MEM) with 5 mM glucose or in hyperglycaemic MEM medium with 25 mM glucose for 24 h or 72 h. Hyperglycaemic MEM medium decreased cell viability compared to control cells cultivated at the physiological glucose level. Moreover, the negative effect of glucose on hepatocyte viability correlated with the duration of administration (Figure 3A). Cells cultivated in hyperglycaemic medium had 72.6 ± 10% viability (24 h), respective 63 ± 8.3% viability (72 h) of control cells cultivated in physiological MEM medium. However, empagliflozin treatment eliminated the toxic effect of glucose at the 25 mM concentration regardless of cultivation time (Figure 3A).

To explore the effect of empagliflozin on lipid storage, hepatocytes were exposed to 1.5 mM oleic acid (OA) in both of MEMs, physiologic or hyperglycaemic. Empagliflozin-treated hepatocytes had lower levels of intracellular lipids compared to non-treated cells (Figure 3B,C). Exposure of cells to glucose or OA contributed to the induction of new senescent cells (Figure 3D,E). However, similarly to *in vivo* experiments, HepG2 cells treated with empagliflozin had reduced senescence-associated beta-galactosidase (SA-β-gal) positivity (Figure 3D,E) as well as mRNA level of the senescent marker *p21^Waf1^* (Figure 3F). Taken together, empagliflozin protected hepatocytes in hyperglycaemic state and led to a reduction in cellular senescence.

### 2.3. Empagliflozin Modulated Metabolomic Profiles in Plasma and Liver

To further explore the effect of empagliflozin on multiple metabolic parameters and more precisely characterise its mode of action NMR-based metabolomic analysis was used. The untargeted multivariate approach was based on the analysis of the whole binned spectra without any previous signal identification. Principal component analysis (PCA) of both plasma and liver samples did not detect any outliers in experimental groups, but they also did not display a clear separation of control and empagliflozin animals (Figures S1 and S2). Nevertheless, certain trends observed in partial least-squares-discriminant analysis (PLS-DA) were evaluated using variable importance in projection (VIP) values obtained for individual bins (PCA and PLS-DA score plots with model validation results; Figures S2 and S3). Identifications of bins with VIP > 1.5 showed which metabolites contributed most to the group separation. A set of eight important metabolites was identified in plasma, namely lactate, glucose, pyruvate, alanine, β-hydroxybutyrate, acetone, leucine and tyrosine. Separation in liver samples was caused mainly by the levels of glycogen, leucine and glutathione. Despite satisfactory cross-validation results, the permutation test showed high P-values, probably indicating overfitting of PLS-DA models. For this reason, the evaluation of significant metabolite changes was based only on the univariate analysis of individual metabolites detected in NMR spectra.

A total of 28 metabolites were identified and quantified in plasma samples, including amino acids, saccharides, nucleosides, organic acids and others (Figure S4). All plasma metabolites significantly affected by empagliflozin administration are summarised in Table 3. The magnitude of treatment effect is presented as a percentage change of normalised concentrations in relation to the control group. Significantly increased levels of leucine and ketone bodies, namely acetone and β-hydroxybutyrate were detected in the empagliflozin-treated group. On the other hand, empagliflozin decreased concentrations of pyruvate, alanine, tyrosine, threonine, cytidine and tryptophan. A total of 32 metabolites were identified and quantified in hydrophilic liver extracts (Figure S5), with significantly changed metabolites reported in Table 4. Significant elevation of glutamine, leucine, valine and uracil was observed in the empagliflozin group. Conversely, a decrease of reduced glutathione, glycogen, and xanthosine levels was induced by empagliflozin administration.

### 2.4. Empagliflozin Improved White Adipose Tissue Insulin Sensitivity

**Figure 4:**
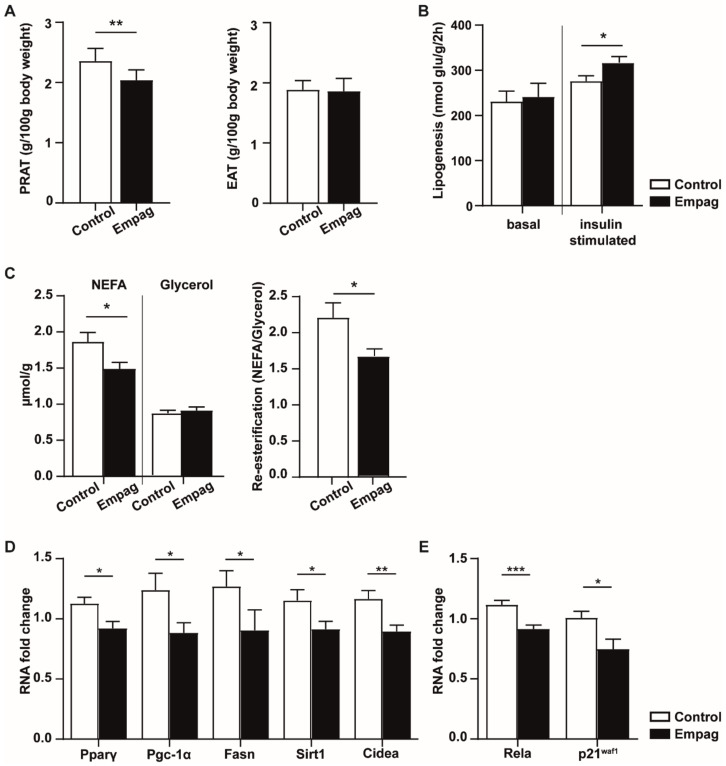
Effect of empagliflozin on white adipose tissue. (**A**) Adiposity is expressed by relative weight to 100 g of body weight of perirenal (PRAT) and epididymal (EAT) adipose tissue mass. (**B**) Lipogenesis: glucose incorporation into epididymal adipose tissue lipids: basal state vs. insulin added into incubation Krebs–Ringer bicarbonate buffer. (**C**) Lipolysis: release of NEFA and glycerol into incubation Krebs–Ringer phosphate buffer; re-esterification: counted as released NEFA/Glycerol ratio. (**D**,**E**) Changes in mRNA level of selected genes involved in lipid metabolism and senescence. Control group (*n* = 7) and empagliflozin group (*n* = 8). Empagliflozin group received a standard diet enriched by 0.01% empagliflozin for 6 weeks. Data are expressed as mean ± SEM; * *p* ˂ 0.05; ** *p* ˂ 0.01; *** *p* ˂ 0.001.

The lower total body weight of the empagliflozin group was most reflected in a decrease of the relative weight of perirenal adipose tissue while no effect was seen on epididymal adipose tissue (EAT) relative weight (Figure 4A). In ex vivo glucose uptake analysis, empagliflozin-treated rats had increased levels of incorporated glucose as compared to the control group suggesting increased insulin sensitivity of EAT (Figure 4B).

As shown in Figure 4C, we observed a decrease in releasing NEFA from EAT in the empagliflozin group, and released glycerol levels were not changed. Decrease of re-esterification (NEFA/Glycerol ratio) in the empagliflozin group indicates that more NEFA were oxidized or re-esterified by EAT than released to buffer.

From the 45 genes measured in EAT (Tables S1 and S2) empagliflozin significantly changed the relative mRNA level of 7 genes (Figure 4D,E), which are mainly involved in (i) energy and lipid metabolism (*Pparγ*: regulating fatty acid storage and activating genes stimulating lipid uptake and adipogenesis by adipocytes; *Pgc-1α*: regulator of mitochondrial biogenesis; *Fasn*: multi-enzyme participating in fatty acid biosynthetic pathway; *Sirt1*: involved in cholesterol metabolism and *Cidea*: playing a role in thermogenesis and lipolysis; Figure 4D) and (ii) cellular senescence (*Rela* and *p21^Waf1^;* Figure 4E). 

To further investigate whether empagliflozin regulates insulin sensitivity and senescence, we used the 3T3-L1 adipocyte cell line, which is used to study SGLT-2 inhibitors [15,16]. The viability of 3T3-L1 cells treated with empagliflozin was measured. We tested empagliflozin at concentrations of 1, 10, 100, and 1000 nM in medium with 25 mM glucose concentration. The viability of 3T3-L1 cells was not affected (Figure S1B), such as in the case of HepG2 cells, and empagliflozin was used at concentration 500 nM for subsequent experiments.

**Figure 5:**
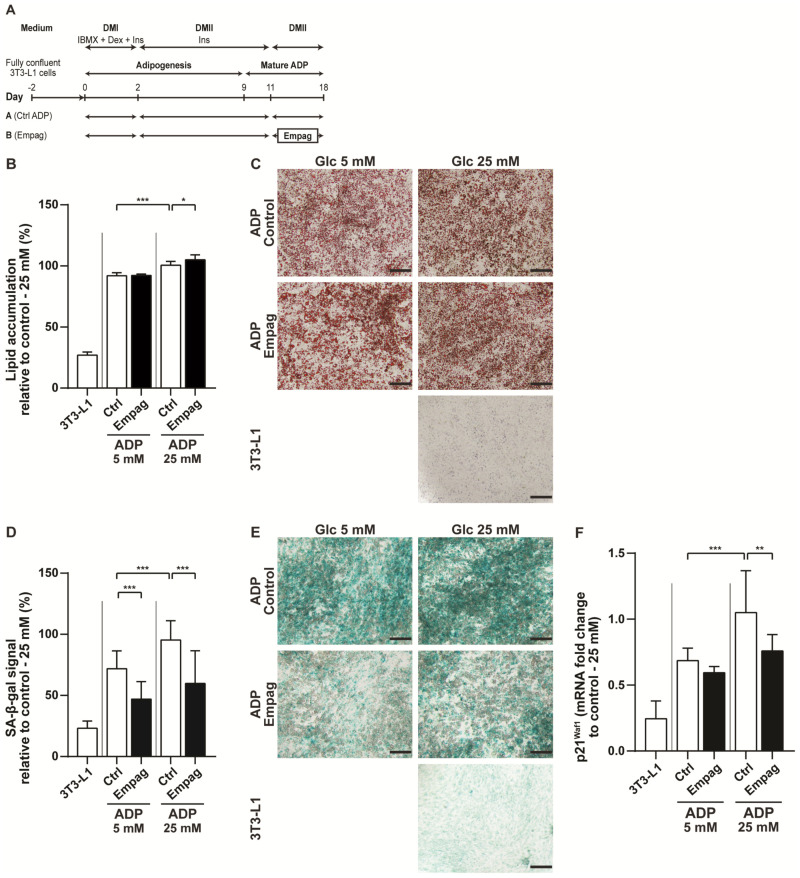
Effect of empagliflozin on mature 3T3-L1 adipocytes. (**A**) Time schedule of the 3T3-L1 preadipocytes (3T3-L1), differentiated into mature adipocytes (ADP) and then exposed to glucose (5 mM or 25 mM) together with or without empagliflozin treatment (500 nM) at time points as indicated. (**B**) Quantification of intracellular lipids accumulation using Oil Red O staining was performed in mature adipocytes and (**C**) representative images are shown. The bar indicates 200 μm. (**D**) Detection of senescent cells evaluated on senescence-associated beta-galactosidase (SA-β-gal) activity and (**E**) representative images are shown. The bar indicates 200 μm. (**F**) Changes of mRNA level of senescence marker *p21^Waf1^*. The results are derived from at least three independent experiments run in triplicates. Data are expressed as mean ± SD; * *p* ˂ 0.05; ** *p* ˂ 0.01; *** *p* ˂ 0.001.

The 3T3-L1 cells can be differentiated into mature adipocytes using a mixture of inducers [17] (Figure 5A). Mature adipocytes were treated with empagliflozin and cultured in physiological Dulbecco’s Modified Eagle Medium (DMEM) with 5 mM glucose or in hyperglycaemic DMEM medium with 25 mM glucose. Adipocytes cultivated in hyperglycaemic medium had a significantly higher amount of intracellular lipids compared to cells cultivated in physiological DMEM (Figure 5B,C). No effect of empagliflozin was observed on mature adipocytes cultivated in physiological DMEM. However, cells cultivated in hyperglycaemic DMEM with empagliflozin had slightly but significantly, increased level of intracellular lipids (Figure 5B,C). Similarly, as in vivo, cells treated with empagliflozin had reduced senescence-associated beta-galactosidase (SA-β-gal) positivity as well as mRNA level of the senescent marker *p21^Waf1^* (Figure 5D–F). These in vitro results support our previous conclusions from in vivo experiments and show that empagliflozin could directly affect adipocyte metabolism.

### 2.5. Empagliflozin Reduced Oxidative Stress and Inflammation in Kidneys

**Figure 6:**
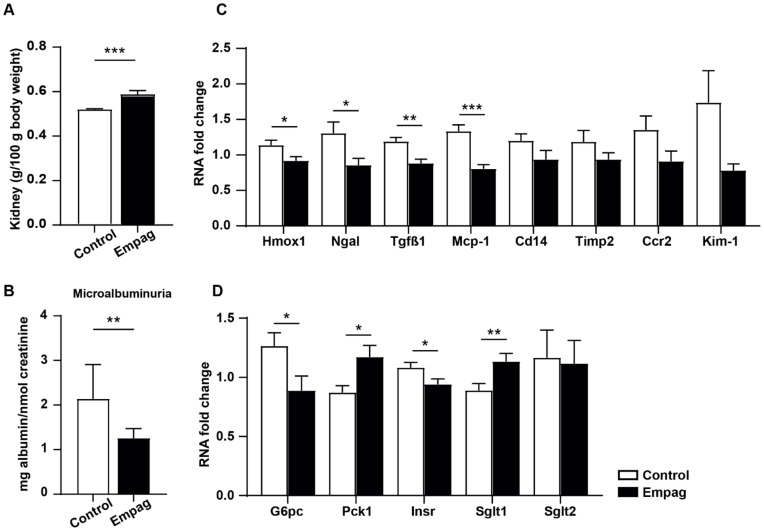
Effect of empagliflozin in the kidney. (**A**) Relative weight is expressed as the weight of kidneys related to 100 g of body weight. (**B**) Microalbuminuria was assessed as albumin/creatinine ratio in urine sample. (**C**) Changes of mRNA level of selected genes involved in inflammatory and injury processes in the kidney cortex. (**D**) Changes of mRNA level of selected genes involved in glucose metabolism in the kidney cortex. Control group (*n* = 7) and empagliflozin group (*n* = 8). Empagliflozin group received a standard diet enriched by 0.01% empagliflozin for 6 weeks. Data are expressed as mean ± SEM; * *p* ˂ 0.05; ** *p* ˂ 0.01; *** *p* ˂ 0.001.

Kidneys are the main target of empagliflozin effects [18]. We observed slightly increased relative kidney weight in the empagliflozin group (Figure 6A). Microalbuminuria expressed as Albumin/Creatinine ratio decreased significantly by 41% after empagliflozin administration (Figure 6B). In the kidney cortex, the activity of the GSH-dependent enzyme, GSH-Px and superoxide dismutase (SOD), an antioxidant enzyme, was increased in the empagliflozin-treated group while levels of lipoperoxidation products measured by TBARS were decreased (Table 5).

In addition to increasing antioxidant enzymes, empagliflozin down-regulated gene expression of nephropathy markers (*Hmox1*, *Ngal*, *Tgfβ1*, *Mcp-1*), which are also involved in inflammatory and injury processes in the kidney cortex. (Figure 6C). *G6pc*, an enzyme involved in gluconeogenesis, and *Insr* were down-regulated by empagliflozin treatment, while the expression of *Pck1*, one of the main gluconeogenic enzymes, was up-regulated. No change in *Sglt2* mRNA level was detected while *Sglt1* expression was up-regulated by empagliflozin administration (Figure 6D).

### 2.6. Empagliflozin Reduced Palmitic Acid Oxidation in Myocardium but Had No Effect on Skeletal Muscle Glucose Metabolism

**Figure 7:**
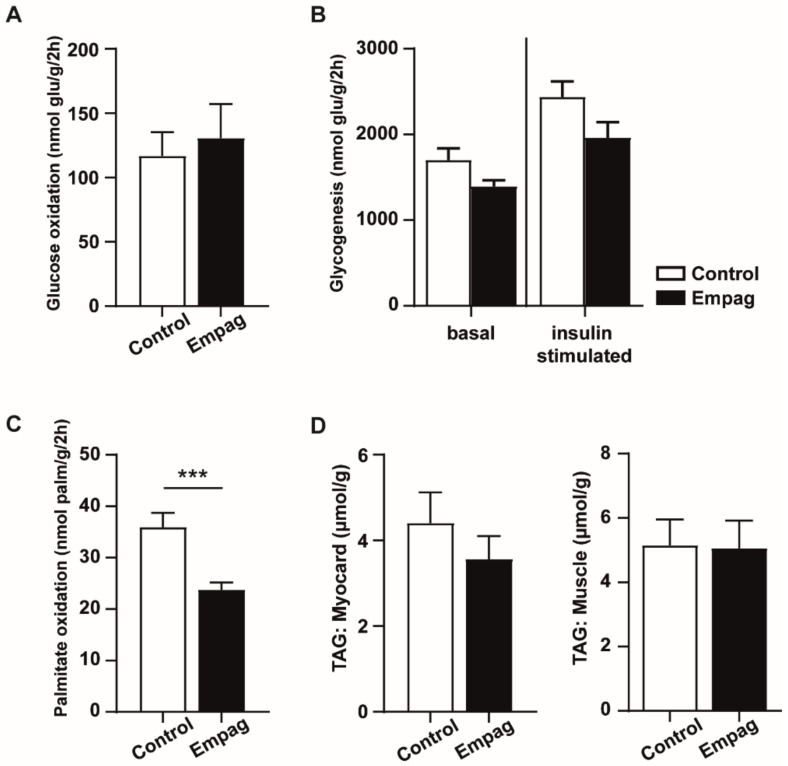
Glucose metabolism in muscle, palmitate oxidation in heart and triglyceride content in muscle and heart. (**A**) Glucose oxidation was measured *ex vivo* by release of CO_2_. (**B**) Glucose incorporation into glycogen of diaphragm. (**C**) Palmitate oxidation measured ex vivo by the release of CO_2_ in myocardium. (**D**) TAG concentrations related to weight of tissue (myocardium and muscle gastrocnemius). Control group (*n* = 7) and empagliflozin group (*n* = 8). Empagliflozin group received a standard diet enriched by 0.01% empagliflozin for 6 weeks. Data are expressed as mean ± SEM; *** *p* ˂ 0.001.

Empagliflozin did not change glucose metabolism in the diaphragm measured by glucose oxidation (Figure 7A) and glucose incorporation into glycogen of muscle tissue (Figure 7B). Moreover, it did not change any of the measured gene expression in soleus muscle (data not shown). In the myocardium, empagliflozin treatment reduced palmitic acid oxidation (Figure 7C). No change in TAG content in skeletal muscle and myocardium was observed (Figure 7D).

## 3. Discussion

Metabolic syndrome, T2DM and obesity are multifactorial diseases associated with many pathologies that collectively increase the risk of cardiovascular or renal events [1,19,20,21]. In addition, obesity and hyperglycaemia contribute to chronic pro-inflammatory conditions, leading to the formation of senescent cells and premature aging of the organism [2,3,4]. SGLT-2 inhibitors, now commonly used in the treatment of type 2 diabetes, show significant positive effects not only on glucose control but also on the prevention and treatment of cardiovascular, renal and heart failure events. Despite numerous positive clinical outcomes, the detailed mechanism of action of SGLT-2 inhibitors on the above-described diseases has not been fully clarified yet [22]. The majority of studies with SGLT-2 inhibitors have been focused on the heart and kidneys, but the major tissues affected by T2DM, liver and adipose tissue, have been explored less often.

In our study, we tested the effect of the SGLT-2 inhibitor empagliflozin on metabolic parameters and insulin resistance using non-obese hHTG rats, a strain characterised by elevated concentrations of TAG; muscle and adipose tissue insulin resistance; hyperinsulinemia and impaired glucose tolerance [23,24,25]. Several studies have demonstrated weight-reducing effects of SGLT-2 inhibitors ipragliflozin [26], dapagliflozin [27] or tofogliflozin [28,29]. Treatment with ipragliflozin in rats with a high-fat diet (HFD)-induced obesity reduced visceral fat mass [26]. In our study, treatment with empagliflozin for six weeks suppressed the increase in body weight despite a slightly higher food intake. The change in body weight was related to reducing fat mass with the most pronounced decrease of perirenal adipose tissue, which is considered a part of visceral adipose tissue. Increased plasma levels of NEFA, together with a decrease of TAG levels observed in the empagliflozin group, may be associated with increased energy requirements due to high urinary glucose loss [30,31]. The shift from carbohydrate oxidation to fatty acid oxidation may lead to the prevention of fat accumulation and inflammation in adipose tissue and liver [29], thus contributing to the improvement of renal and cardiovascular outcomes in the long-term run. In our model of metabolic syndrome, we observed a reduction of senescent markers together with an increase of HDL-C and improved insulin sensitivity of EAT. A similar effect was observed for 3T3-L1 mature adipocytes treated with empagliflozin. During adipogenesis under hyperglycemic conditions, 3T3-L1 adipocytes became insulin resistant [32]. Higher content of intracellular lipids in 3T3-L1 adipocytes under hyperglycaemic conditions after empagliflozin treatment might be explained as improved insulin sensitivity of the mature adipocytes and thus increased glucose incorporation into cells.

Lower NEFA/Glycerol ratio release during lipolysis in EAT indicates a shift from glucose to lipid oxidation, also observed in humans [33] treated with dapagliflozin. In patients with T2DM treated with empagliflozin, levels of HDL-cholesterol significantly increased and LDL-cholesterol and TAG levels decreased while being unaffected in the placebo group [34]. Thus in our experimental model, empagliflozin administration improved lipid and glucose metabolism and eliminated multiple risk factors associated with the development of insulin resistance, diabetes and its complications [35].

Excessive lipid content in the liver, commonly referred to as non-alcoholic fatty liver disease (NAFLD), may further progress to non-alcoholic steatohepatitis (NASH), which is a more severe form of liver disease characterised by hepatocyte injury, inflammation, and fibrosis and by association with a higher risk of cardiovascular diseases [36]. There is evidence that HepG2 cells cultivated in hyperglycaemic media have elevated lipid content together with increased gene expression of key biomarkers of insulin resistance or NAFLD [37]. Reduction of intracellular lipid accumulation after empagliflozin treatment in our study suggests its protective effect on HepG2 cells. Increased pro-inflammatory state in the liver is among other factors driven by senescence cells secreting pro-inflammatory cytokines (SASP), leading to mitochondrial dysfunction, increased oxidative stress [38] and decreased ability of hepatocytes to metabolize fatty acids efficiently [39]. Decreased gene expression of senescent marker *p21^Waf1^*, reduction of oxidative stress and increased fatty acid oxidation observed in the empagliflozin group in our study suggest the improvement of numerous factors involved in the development of NAFLD or NASH. Furthermore, we observed increased expression of liver genes linked to stimulation of fatty acid oxidation such as Carnitine palmitoyl transferase 1 (*Cpt1*) together with down-regulated gene expression of Fatty acid synthase (*Fasn*) commonly up-regulated in NAFLD [40], indicating protective effects of empagliflozin in the liver. We also demonstrated a reduction of senescent marker *p21^Waf1^* along with lower fat accumulation in the empagliflozin-treated HepG2 hepatocytes. Similar results were obtained previously by other groups for the HepG2 cell line using other SGLT-2 inhibitors. Dapagliflozin ameliorated hepatic steatosis in Zucker diabetic fatty (ZDF) rats and palmitic acid-stimulated LO2 cells and HepG2 cells [14], while canagliflozin showed similar effects in a mouse model of diabetes and NASH-related hepatocellular carcinoma [41], leading the authors to suggest the use of dapagliflozin in the treatment of NASH or NAFLD [14,41]. Interestingly, in higher concentrations, (10 µM) canagliflozin also protected against liver carcinogenesis [41,42]. Some of the novel SGLT inhibitors, such as tofogliflozin [43] or trilobatin [44], also promoted liver proliferation. These results indicate an overall positive effect of SGLT inhibitors in the liver, and our data add another important piece of information with the potential role of empagliflozin in the amelioration of liver cell senescence.

In addition to the presence of fatty liver disease, a close association has been described between metabolic syndrome and the risk of developing renal damage [45]. There is also a connection of the presence of metabolic syndrome and higher urinary albumin excretion in non-diabetic, essential hypertensive patients [46]. Chronic hyperglycaemia and dyslipidaemia induce mitochondrial dysregulation, oxidative stress [47] and inflammation [48,49], which can be ameliorated by pleiotropic effects of empagliflozin [50]. The decrease of albuminuria after empagliflozin treatment in our study confirms its nephroprotective effect. Gene expression analysis in the kidney showed decreased levels of *Tgfβ1*, a key pro-fibrotic mediator [51], along with lower gene expression of heme oxygenase 1 (*Hmox1*), an adaptive responder to a wide variety of stimulators of oxidative stress (including heme, H_2_O_2_, cytokines, growth factors, heavy metals, nitric oxide or oxidized LDL) [52,53] and *Ngal*, a member of the lipocalin family, that is significantly expressed in injured epithelial cells [54]. Interestingly, despite the above described nephroprotective effects, kidney weights of empagliflozin-treated animals increased, which is in agreement with the results of other studies in non-diabetic mice with *Sglt2* knockout [55] and empagliflozin treated high-fat-diet-induced obese (DIO) mice [56]. Moreover, empagliflozin increased gene expression of phosphoenolpyruvate carboxykinase 1 (*Pck1*), a gluconeogenic enzyme, along with decreased gene expression of insulin receptor (*Insr*). Both of these parameters were in previous studies associated with metabolic syndrome and/or insulin resistance in HFD C57BL/6 mice and human proximal tubule cells, respectively [57]. While under diabetic conditions, the amount of SGLT-2 in proximal tubules increases [58], in our non-diabetic rat model the expression of *Sglt2* was unchanged while the expression of *Sglt1* was increased. The compensatory increase of SGLT-1 was previously described after genetic or pharmacological inhibition of SGLT-2 [59]. 

Urinary glucose excretion leads to adaptive responses in glucose homeostasis. Glycosuria induction following SGLT-2 inhibition is associated with a paradoxical increase in endogenous glucose production in mice and humans [60,61]. In the liver, we observed a decrease in glycogen, the substrate for glycogenolysis (a metabolic pathway of endogenous glucose production) together with increased PPARγ coactivator 1α (*Pgc-1α*) and phosphoenolpyruvate carboxykinase 1 (*Pck1*), which induces hepatic glucose production [62].

In our study, NMR-based metabolomics revealed changes in metabolites in glucose metabolic pathways, indicating several compensatory mechanisms to urinary glucose loss. Branched-chain amino acids (BCAA), namely leucine both in plasma and liver and valine in the liver were increased. Interestingly, higher leucine and valine levels were previously associated with a lower risk of cardiovascular mortality in individuals with T2DM [63]. However, the relationship between BCAA, glucose metabolism and insulin resistance is not fully clarified [64,65]. It was proposed that under protein catabolism, BCAA can be used for the enhanced production of alanine and glutamine [66], which are the most crucial glucose precursors [67]. The decrease of pyruvate by empagliflozin treatment in our study can be caused by attenuated glycolysis and is in accordance with the observed lower mRNA level of glucose-6-phosphatase (*G6pc*) in the kidney cortex. Moreover, we also detected a decreased level of alanine, which can be caused by its consumption of gluconeogenesis [68]. The increased demand for gluconeogenesis in our study was accompanied by increased glutamine formation, which was also observed in empagliflozin-treated Otsuka Long-Evans Tokushima Fatty (OLETF) rats in the myocardium [69].

In summary, our study identified numerous complex effects of empagliflozin with a possible contribution to overall cardio-renal protection in the long-term run. In addition to improved parameters of oxidative stress in the liver and kidneys, decreased markers of renal injury, lower fat accumulation and improvement of insulin sensitivity in fat, our data suggest that the elimination of senescent cells and their SASP may also contribute to positive metabolic, cardio- and reno-protective effects of empagliflozin.

## 4. Materials and Methods

### 4.1. Animals and Diets

In our study, we used hHTG rats as a model of metabolic syndrome disturbances. This strain is characterised by elevated circulating TAG concentrations and their ectopic accumulation in the liver and muscle; muscle and adipose tissue resistance to insulin; hyperinsulinemia and impaired glucose tolerance. Detailed characteristics of this rat strain were already described in previous studies [23,24,25].

Male rats were fed a standard diet (23% proteins, 43% starch, 7% fat, 5% fibre, and 1% vitamin and mineral mixture, Bonagro, Blazovice, Czech Republic). Eight months old rats were randomly divided into 2 groups as follows: control (*n* = 7) and empagliflozin (*n* = 8). Empagliflozin group received a standard diet enriched by 0.01% empagliflozin (Boehringer Ingelheim Pharma GmbH&Co. KG, Ingelheim am Rhein, Germany) equivalent to 10 mg/kg body weight. The empagliflozin dose was based on previously published studies [70,71,72]. The rats were treated for 6 weeks before the tissues from rats were collected and measurements were performed. The rats were housed in an air-conditioned animal facility and were allowed free access to chow and water. At the end of the experiments, animals were sacrificed between 9am and 11am by decapitation in a postprandial state, and blood and tissues were collected for further analyses. Experiments were performed in agreement with the Animal Protection Law of the Czech Republic (53/2018) and were approved by the Ethics Committee of the Institute for Clinical and Experimental Medicine.

### 4.2. Body Weight, Food and Water Consumption

Body weight was measured once a week during 6 weeks of empagliflozin treatment. Food consumption was measured 3 times a week and water consumption 2 times a week. Daily food intake was measured as a food intake by cage (*n* = 3–4) and calculated by averaging for 1 animal/day over the entire course of the study.

### 4.3. Urinary Glucose and Microalbuminuria

Rats were housed individually in metabolic cages for 24 h and urine samples were collected in glass tubes. Glucose levels were measured by glucose oxidase assay (GLU GOD, Erba-Lachema, Brno, Czech Republic). The level of albumin in urine was analysed by high-performance liquid chromatography with UV-VIS detection described in detail in [73] and was adjusted for creatinine concentration. The presence of microalbuminuria was evaluated by measuring the albumin to creatinine ratio (A/C).

### 4.4. Biochemical Analyses

Plasma glucose levels were measured by glucose oxidase assay (GLU GOD, Erba-Lachema, Brno, Czech Republic) and TAG, cholesterol, HDL-cholesterol (Erba-Lachema, Brno, Czech Republic), and glycerol (Randox Laboratories Ltd., Crumlin, UK) concentrations were quantified using standard enzymatic methods. Non-esterified fatty acid levels (NEFA) were determined using an acyl-CoA oxidase-based colorimetric kit (Roche Diagnostics GmbH, Mannheim, Germany). Plasma insulin concentrations were measured using a rat insulin enzyme-linked immunosorbent assay kit (Mercodia, Uppsala, Sweden). MCP-1 and leptin concentrations were measured by rat multiplex enzyme-linked immunosorbent assay kit (Milliplex: RADPKMAG-80K, Merck KGaA, Darmstadt, Germany).

### 4.5. Glucose Utilization in White Adipose Tissue, Glucose Oxidation and Glycogen Synthesis in Diaphragm

Glucose utilization in isolated epididymal adipose tissue was measured by incorporation of ^14^C-U glucose into adipose tissue lipids. Glucose oxidation was determined in isolated diaphragmatic muscle by measuring the incorporation of ^14^C-U glucose into CO_2_. For the measurement of glycogen content, the diaphragm after first incubation was rinsed in saline and immediately put into chloroform:methanol (2:1). All these methods were described in detail previously [74].

### 4.6. Palmitic Acid Oxidation in Isolated Myocardium

Palmitic acid utilization in myocardium was determined *ex vivo* by measuring ^14^C-palmitic acid oxidation to CO_2_. The removed left ventricle was immediately sliced and incubated for 2 h in 5 mL of Krebs–Ringer bicarbonate buffer, pH 7.4. The buffer contained 1 mM unlabelled palmitate, 18.5 kBq/mL of ^14^C-palmitate (Perkin Elmer, Waltham, Massachusetts, USA) and 3 mg/mL BSA fraction V (Sigma-Aldrich, St. Louis, MO, USA). The incubation was performed in a 95% O_2_ + 5% CO_2_ atmosphere at 37 °C in sealed vials in a shaking water bath. After incubation, myocardium slices were removed and 0.2 mL of 1 M hyamine hydroxide was injected into the central compartment of the incubation vial and 0.5 mL of 0.5 M H_2_SO_4_ was added to the main compartment to liberate CO_2_. These sealed vessels were incubated for another 45 min in an air atmosphere. The hyamine hydroxide with absorbed CO_2_ was then quantitatively transferred into a scintillation vial containing 10 mL scintillation fluid (Rotiszint, Carl Roth GmbH + Co. KG, Karlsruhe, Germany) for radioactivity counting.

### 4.7. Lipolysis in Isolated Epididymal Adipose Tissue

The measurement of lipolysis was performed as described in detail previously [75]. Briefly, the tissue was incubated in the Krebs–Ringer phosphate buffer for 2 h and the concentrations of NEFA and glycerol released from tissues in the medium were measured using an acyl-CoA oxidase-based colorimetric kit for NEFA (Roche Diagnostics GmbH, Mannheim, Germany) and standard enzymatic method for glycerol (Randox Laboratories Ltd., Crumlin, County Antrim, UK).

### 4.8. Tissue Triglyceride Measurements

For determination of TAG in liver, heart and musculus gastrocnemius, tissues were homogenised, and TAG was extracted for 16 h in chloroform:methanol (2:1), then 2% KH_2_PO_4_ was added. After 24 h the organic phase was removed and evaporated. The resulting pellet was dissolved in isopropyl alcohol and TAG content was determined by enzymatic assay (TG L 250 S, Erba-Lachema, Brno, Czech Republic).

### 4.9. Anthrone Method (Polysacharide Quantification): Liver Glycogen

For measurement of glycogen concentration, part of the liver (50–100 mg) was put into pre-cooled 30% KOH (1 mL) immediately after dissection, than tissue was disintegrated by boiling for 1 h and washed with 96% ethanol. After 24 h the samples were centrifuged and the pellet was dissolved in H_2_O and 96% ethanol, samples were heated up till boiling point. After 2.5 h the samples were centrifuged, and the pellet was dissolved in H_2_O. For polysaccharide quantification, the Anthrone method was used. 1 mL of dissolved extract was mixed with 5 mL of the Anthrone reagent (50 mg Anthrone + 79 mL 95% H_2_SO_4_ + 31 mL H_2_O) and boiled for 13 min. Absorbance (600 OD) of the samples and standard (GLU GOD, Erba-Lachema, Brno, Czech Republic) was measured and concentration counted by linear regression.

### 4.10. Oxidative Stress Parameters

Activities of antioxidant enzymes, superoxide dismutase (SOD), glutathione peroxidase (GSH-Px) and glutathione reductase (GR) were analysed using Cayman Chemicals assay kits (Ann Arbor, Michigan, USA). Catalase (CAT) activity was determined based on the ability of H_2_O_2_ to form a colour complex with ammonium molybdate, and with spectrophotometric detection. Concentrations of conjugated dienes (CD) were determined by extraction in media (heptane:isopropanol = 2:1) and measured spectrophotometrically in the heptane layer. Lipoperoxidation products were analysed based on levels of thiobarbituric acid-reactive substances (TBARS) [76]. All parameters were adjusted to the tissue protein concentration and plasma volume.

### 4.11. Isolation and Determination of mRNA Level: TLDA (TaqMan Low Density Array)

Samples of EAT, liver and kidney were snap-frozen in liquid nitrogen. Tissues were homogenised on MagNA Lyser Instrument with MagNA Lyser Green beads (Roche Diagnostics GmbH, Mannheim, Germany). Total RNA from homogenised tissue was extracted on MagNA Pure instrument using Magna Pure Compact RNA Isolation kit (tissue) (Roche Diagnostics GmbH, Mannheim, Germany). The RNA concentration was determined from absorbance at 260 nm on a NanoPhotometer (Implen, München, Germany). Reverse transcription was performed using 0.25 μg of total RNA to synthesise the first-strand cDNA using the random primers as per the instructions of the High-Capacity cDNA Reverse Transcription Kit (Applied Biosystems, Waltham, Massachusetts, USA). TaqMan Array Custom Micro Fluidic Card with predesigned specific TaqManGene Expression Assays (Applied Biosystems, Waltham, Massachusetts, USA) was used for the reaction. Beta-2 microglobulin (*B2m*) was used as an endogenous reference. The formula 2^−ΔΔCt^ was used to calculate relative gene expression [77]. The list of measured and control genes is shown in Tables S1 and S2.

### 4.12. NMR-Based Metabolomics in Plasma and Liver Samples

Proteins were precipitated from plasma samples using methanol; vacuum-dried supernatants were dissolved in D_2_O with phosphate buffer (pH 7.4) and transferred into 3 mm NMR tubes. Liver samples were extracted with formate buffer (pH 2.75), any protein residues were precipitated with methanol; vacuum-dried supernatants were dissolved in D_2_O with phosphate buffer (pH 7.4) and transferred into 5 mm NMR tubes. The detailed protocol of the sample preparation is described in Text S1.

The NMR data were acquired at 298 K on a 600 MHz Bruker Avance III spectrometer (Bruker BioSpin, Rheinstetten, Germany) equipped with a 5 mm TCI cryogenic probe head. The proton spectra were acquired by a Carr-Purcell-Meiboom-Gill (CPMG) pulse program with presaturation during relaxation delay; this pulse sequence suppresses broad signals of residual high-molecular-weight compounds and thus provides a flatter baseline. Additionally, a short *J*-resolved experiment with presaturation was recorded for each sample to partially solve the problem with the signal overlap. The detailed setting of NMR experiments is described in Text S2. The raw spectral data were processed using TopSpin 3.5 software (Bruker BioSpin, Ettlingen, Germany), statistical analysis was carried out using MetaboAnalyst 3.6 [78] and MATLAB software (The MathWorks, Inc., Natick, MA, USA).

After referencing the signal of TSP = 0.00 ppm, all spectra were evaluated in the range 0.2–10.0 ppm. Spectral regions with signals of water, urea, methanol, EDTA in plasma samples and water, methanol and formate in liver extracts were excluded before the following statistical analysis. All spectra were normalised using the probabilistic quotient normalization (PQN) method [79] with a pooled control group as a reference.

### 4.13. Cell Culture Analyses

Mouse 3T3-L1 preadipocytes were purchased from the American Type Culture Collection and maintained in Dulbecco’s modified Eagle’s medium (DMEM) containing 4.5 g/L d-glucose and 1 mM sodium pyruvate (Gibco, Grand Island, NY, USA), supplemented with 10% foetal bovine serum (Gibco, Grand Island, NY, USA), 4 mM l-glutamine (Gibco, Grand Island, NY, USA), and the mixture of 100 U/mL penicillin and 100 μg/mL streptomycin sulphate (Sigma-Aldrich, St. Louis, MO, USA). The cells were differentiated into mature adipocytes, as described previously [17,80], by the addition of differentiation mixture 0.5 mM 3-isobutyl-1-methylxanthine (IBMX; Sigma-Aldrich, St. Louis, MO, USA), 0.4 µM dexamethasone (Dex; Sigma-Aldrich, St. Louis, MO, USA), 1.7 µM insulin (Ins; figure) and 25 mM HEPES (Sigma-Aldrich, St. Louis, MO, USA). 3T3-L1 cells were differentiated into mature adipocytes for 11 days.

Human HepG2 hepatocytes were purchased from the American Type Culture Collection and maintained in Minimum Essential Medium (MEM) containing 1 g/L or 4.5 g/L d-glucose (Gibco, Grand Island, NY, USA) and supplemented with 10% foetal bovine serum (Gibco, Grand Island, NY, USA), 2 mM l-glutamine (Gibco, Grand Island, NY, USA), non-essential amino acids (Gibco, Grand Island, NY, USA) and mixture of 100 U/mL penicillin and 100 μg/mL streptomycin sulphate (Sigma-Aldrich, St. Louis, MO, USA). For induction of steatosis, HepG2 cells were seeded into 12-well (75 × 10^3^ cells per well). The next day, HepG2 cells were exposed to standard MEM supplemented with 1.5 mM oleic acid (OA; Sigma-Aldrich, St. Louis, MO, USA) for 24 h. For all experiments, 3T3-L1 cells and HepG2 cells were treated in medium with 500 nM empagliflozin (Boehringer Ingelheim International GmbH, Ingelheim am Rhein, Germany). The cells were kept at 37 °C under 5% CO_2_ in a humidified atmosphere.

### 4.14. Detection of Senescence-Associated β-Galactosidase Activity

Mature 3T3-L1 adipocytes or HepG2 hepatocytes were washed twice with phosphate buffer. Cells were fixed with 0.5% glutaraldehyde in phosphate buffer for 10 min and then washed two times with phosphate buffer (pH 5.5) supplemented with 1 mM MgCl_2_. The cells were stained with the X-gal solution (1 mg/mL X-gal, 0.12 mM K_3_Fe(CN)_6_, 0.12 mM K_4_Fe(CN)_6_, 1 mM MgCl_2_ in phosphate buffer at pH 5.5) for 5 h in the dark at 37 °C. After washing 3 times with phosphate buffer, blue signal of senescence-associated beta-galactosidase (SA-β-gal) was detected using a light microscope (Olympus IX83, Tokyo, Japan) and evaluated using the ImageJ 1.52v software (National Institutes of Health, Bethesda, MD, USA). Due to high cell numbers, the intensity of the blue senescence-associated beta-galactosidase (SA-β-gal) signal was evaluated for the whole image and related to control.

### 4.15. Isolation and Quantification of Gene Expression in Cell Lines

Total RNA from HepG2 hepatocytes and 3T3-L1 adipocytes was isolated using RNeasy Mini Kit (Qiagen, Germantown, MD, USA). The amount of 1 µg of total RNA was used to reverse transcription using FIREScript^®^ RT cDNA synthesis MIX (Solis BioDyne, Tartu, Estonia) with random primers. cDNA solution in volume 3 µL was used for quantitative PCR using HOT FIREPol^®^ EvaGreen^®^ qPCR Supermix (Solis BioDyne, Tartu, Estonia) with concentration of primers 1 µM. RT-qPCR reaction run on a CFX96 Touch™ (Bio-Rad, Hercules, CA, USA). The relative quantity of cDNA was estimated by the 2^−ΔΔCt^ method, and data were normalised to Beta-2 microglobulin (*B2m*) [81,82]. Primers were purchased from Metabion (Metabion, Planegg/Steinkirchen, Germany) and sequences are described in Table S3.

### 4.16. Oil Red O Staining

Lipid droplets in mature 3T3-L1 adipocytes or HepG2 hepatocytes were visualized and quantified by Oil Red O staining, as previously described [17,83] with slight modifications. The cells were fixed with 3.7% formaldehyde (P-LAB, Prague, Czech Republic) in phosphate buffer (pH 7.4) for 30 min and stained with freshly prepared 0.3% Oil Red O staining solution (Sigma-Aldrich, St. Louis, MO, USA) in isopropanol for 40 min. The cells were then washed 5 times with distilled water. The signal of Oil Red O in lipid droplets was observed and imaged using light microscopy (Olympus IX83, Tokyo, Japan) at 200× magnification. For quantification, the Oil Red O dye was extracted into 100% isopropanol for 10 min at room temperature. Absorption of Oil red O dye was measured at 500 nm.

### 4.17. Cell Viability

The cells were seeded into 96 well (5 × 10^3^ cells per well for HepG2 cells and 2 × 10^3^ cells per well for 3T3-L1 cells). The effect of empagliflozin on HepG2 and 3T3-L1 cell viability was determined by 24 h cultivation of cells with 1, 10, 100 and 1000 nM concentrations of empagliflozin. The cell viability was determined using Cell Proliferation Reagent WST-1 (Roche Diagnostics GmbH, Mannheim, Germany).

The effect of glucose on HepG2 cell viability was determined by 24 h and 72 h cultivation of cells with 1 g/L (5 mM) or 4.5 g/L (25 mM) concentration of glucose (Sigma-Aldrich, St. Louis, MO, USA). The cell viability was determined using Cell Proliferation Reagent WST-1 (Roche Diagnostics GmbH, Mannheim, Germany).

### 4.18. Statistical Analysis

The data were expressed as means ± SEM. Individual groups were compared by unpaired t-test using the GraphPad Prism software (GraphPad Software Inc., San Diego, CA, USA). Statistical significance was set as *p* < 0.05.

Metabolomic data were evaluated using both targeted and untargeted approaches. For untargeted multivariate analysis, normalised spectra were binned to 0.01 ppm intervals and Pareto scaled. Principal component analysis (PCA) was performed to detect possible outliers and display trends in sample grouping. Next, the partial least-squares-discriminant analysis (PLS-DA) was applied to find signals responsible for the group separation. PLS-DA models were validated by leave-one-out cross-validation and the permutation test. The results of the PLS-DA models aggregated in variable importance in projection (VIP) scores were used for identification of which bins contributed the most to the differentiation between empagliflozin-treated and control rats.

Furthermore, metabolomic profiling using univariate statistics was applied. Individual signals detected in spectra were identified using the Chenomx NMR Suite 7.5 database (Chenomx Inc., Edmonton, AB, Canada) and the Human Metabolome Database (HMDB) [84]. Based on the Lilliefors test of normality, normalised signals intensities were subjected to the unpaired Student t-test to determine the statistical significance (defined as *p* < 0.05) of the observed changes in metabolite concentrations.

## Figures and Tables

**Table 1:** Biochemical parameters in hHTG rats treated with empagliflozin compared to control.

Parameter	Units	Control	Empagliflozin
Urinary glucose	mmol/L	0.9 ± 0.2	93.7 ± 12.2 ***
Glucose fasted	mmol/L	5.314 ± 0.347	4.407 ± 0.131 *
Glucose postprandial	mmol/L	8.296 ± 0.347	7.969 ± 0.248
Insulin	nmol/L	0.221 ± 0.024	0.142 ± 0.024 *
TAG	mmol/L	4.750 ± 0.294	3.12 ± 0.226 ***
CHOL	mmol/L	1.567 ± 0.014	1.536 ± 0.032
HDL-C	mmol/L	0.760 ± 0.021	0.913 ± 0.037 **
NEFA	mmol/L	0.426 ± 0.027	0.666 ± 0.083 **
Glycerol	mmol/L	0.192 ± 0.013	0.203 ± 0.013
Leptin	pg/mL	4425 ± 220	3508 ± 245 *
MCP-1	pg/mL	149.62 ± 67.25	123.1 ± 15.5

Data are expressed as mean ± SEM; * *p* ˂ 0.05, ** *p* ˂ 0.01, *** *p* ˂ 0.001. TAG: plasma triglycerides; CHOL: plasma cholesterol; HDL-C: HDL cholesterol; NEFA: non-esterified fatty acids; MCP-1: monocyte chemoattractant protein 1. Control group (*n* = 7) and empagliflozin group (*n* = 8). Empagliflozin group received standard diet enriched by 0.01% empagliflozin for 6 weeks.

**Table 2:** Biochemical parameters in hHTG rats treated with empagliflozin compared to control.

Parameter	Units	Control	Empagliflozin
SOD	U/mg	0.139 ± 0.008	0.126 ± 0.006
GSH-Px	µM NADPH/min/mg	322 ± 21	380 ± 17 *
GR	nM NADPH/min/mg	87 ± 4	101 ± 3 **
CAT	µM H_2_O_2_/min/mg	1556 ± 194	2074 ± 110 *
CD	nM/mg	36.9 ± 1.6	41.1 ± 3.3
TBARS	nM/mg	1.91 ± 0.12	1.50 ± 0.11 *

Data are expressed as mean ± SEM; * *p* ˂ 0.05, ** *p* ˂ 0.01. SOD: superoxide dismutase; GSH-Px: glutathione peroxidase; GR: glutathione reductase; CAT: catalase; CD: conjugated dienes; TBARS: thiobarbituric acid reactive substances. Control group (*n* = 7) and empagliflozin group (*n* = 8). Empagliflozin group received standard diet enriched by 0.01% empagliflozin for 6 weeks.

**Table 3:** Significantly changed metabolites in plasma.

Metabolite	NMR Signal Used for Quantitation [ppm]	(Empag-Control)/ Control [%]	*p*-Value
Acetone	2.24 (s)	138.5	0.002
β-Hydroxybutyrate	1.20 (d)	66.7	0.043
Leucine	0.97 (m)	12.6	0.028
Pyruvate	2.38 (s)	−33.3	0.007
Alanine	1.49 (d)	−14.2	0.003
Tyrosine	6.91 (m)	−20.0	0.034
Threonine	4.25 (dd)	−11.5	0.008
Cytidine	6.07 (d)	−9.6	0.021
Tryptophan	7.29 (m)	−8.1	0.058

Data are expressed as the percentage change of normalised concentrations in empagliflozin versus control groups. Statistical significance was determined by the unpaired Student *t*-test. Control group (*n* = 7) and empagliflozin group (*n* = 8). Empagliflozin group received a standard diet enriched by 0.01% empagliflozin for 6 weeks. Signal multiplicity is marked as follows: (s)-singlet, (d)-doublet, (dd)-doublet of doublets, (m)-multiplet.

**Table 4:** Significantly changed metabolites in the liver extracts.

Metabolite	NMR Signal Used for Quantitation [ppm]	(Empag-Control)/ Control [%]	*p*-Value
Glutamine	2.45 (m)	23.6	0.014
Leucine	1.71 (m)	12.3	0.032
Valine	0.99 (d)	10.6	0.028
Uracil	7.54 (d)	11.5	0.017
Glutathione (reduced)	4.57 (dd)	−43.1	0.020
Glycogen	5.41 (m)	−22.0	0.001
Xanthosine	5.86 (d)	−18.9	0.027

Data are expressed as the percentage change of normalised concentrations in empagliflozin versus control groups. Statistical significance was determined by the unpaired Student *t*-test. Control group (*n* = 7) and empagliflozin group (*n* = 8). Empagliflozin group received a standard diet enriched by 0.01% empagliflozin for 6 weeks. Signal multiplicity is marked as follows: (s)-singlet, (d)-doublet, (dd)-doublet of doublets, (m)-multiplet.

**Table 5:** Parameters of oxidative stress in kidney cortex.

Parameter	Units	Control	Empagliflozin
SOD	U/mg	0.067 ± 0.01	0.088 ± 0.01 *
GSH-Px	µM NADPH/min/mg	128 ± 12	186 ± 15 **
GR	nM NADPH/min/mg	54.4 ± 3.2	57.3 ± 3.7
CAT	µM H_2_O_2_/min/mg	17.7 ± 1.42	20.1 ± 1.09
CD	nM/mg	24.3 ± 1.34	22.3 ± 1.93
TBARS	nM/mg	0.681 ± 0.02	0.560 ± 0.03 **

Data are expressed as mean ± SEM; * *p* < 0.05, ** *p* < 0.01. Statistical significance was determined by the unpaired Student t-test. SOD: superoxide dismutase; GSH-Px: glutathione peroxidase; GR: glutathione reductase; CAT: catalase; CD: conjugated dienes; TBARS: thiobarbituric acid reactive substances. Control group (*n* = 7) and empagliflozin group (*n* = 8). Empagliflozin group received standard diet enriched by 0.01% empagliflozin for 6 weeks.

## Data Availability

The data presented in this study are available on request from the corresponding authors.

## Supplementary Materials

Supplementary Materials can be found at www.mdpi.com/1422-0067/221/91/606/s1.
