# Supplementary material for: Complex Positive Effects of SGLT-2 Inhibitor Empagliflozin in the Liver, Kidney and Adipose Tissue of Hereditary Hypertriglyceridemic Rats: Possible Contribution of Attenuation of Cell Senescence and Oxidative Stress
Source: Int J Mol Sci. 2021 Sep 30;22(19):10606. doi: 10.3390/ijms221910606 (PMC8508693; doi:10.3390/ijms221910606)
Supplement: Supplementary file 1 [file ijms-22-10606-s001.zip › ijms-1386978-supplementary.pdf]

Supplementary Materials

Supporting Text

Text S1. Sample preparation for NMR-based metabolomics

145 µl plasma samples were mixed with 290 µl cold methanol, vortexed, and incubated at -20 °C for 30 min. The mixture was centrifuged at 14.000 rpm for 30 min at 4 °C; 360 µl supernatant were vacuum-dried. Dry matter was mixed with 162 µl D_2O and 18 µl phosphate buffer (1.5 M, pH = 7.4, in D_2O, containing 0.58 mM TSP) and transferred to 3 mm NMR tubes. Approximate 90 mg liver tissue was vortexed with 540 µl formate buffer (0.4 M, pH = 2.75), than cold methanol in a ratio 1:4 (v/v) was added and the sample was left at -80 °C overnight. The mixture was centrifuged at 9.000 rpm for 30 min at 4 °C; 1250 µl supernatant were vacuum-dried. Dry extract was mixed with 450 µl D_2O and 50 µl phosphate buffer (1.5 M, pH = 7.4, in D_2O, containing 0.58 mM TSP) and transferred to 5 mm NMR tubes.

Text S2. NMR experiments

The NMR spectra were recorded at 298 K on a 600 MHz Bruker Avance III spectrometer (Bruker BioSpin, Rheinstetten, Germany) equipped with a 5-mm TCI cryogenic probe head. Tuning, matching, shimming and adjusting of the 90° pulse length were carried out for each sample in automatic regime.

The proton spectra were acquired by a Carr-Purcell-Meiboom-Gill (CPMG) pulse program (cpmgpr1d) with presaturation during relaxation delay (4 s) using a 25-Hz saturation pulse centered on the water resonance; number of scans (NS) = 128; number of data points (TD) = 64 k; spectral width (SW) = 20 ppm; echo time = 0.3 ms; loop for T2 filter = 126. Additionally, a short J-resolved experiment with presaturation (jresgpprqf, NS = 4, SW = 16 ppm, TD = 8 k, number of increments = 40, SW = 78.125 Hz in the indirect dimension, and relaxation delay = 2 s) was recorded for each sample to partially solve the problem with the signal overlap.

Supporting figures

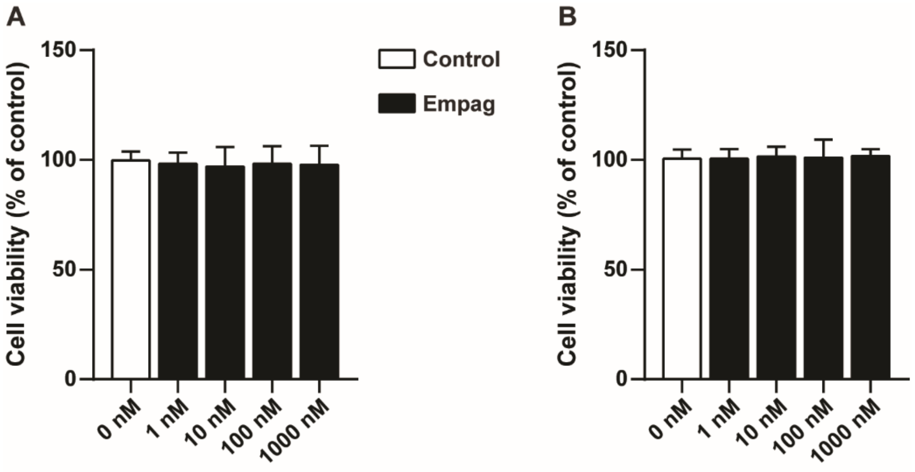

Figure S1. Effect of empagliflozin on the viability of HepG2 and 3T3-L1 cell lines. (**A**) HepG2 cells were cultivated in MEM with 5 mM glucose and empagliflozin (0 – 1000 nM) for 24 h. (**B**) 3T3-L1 cells were cultivated in DMEM with 25 mM glucose and empagliflozin (0 – 1000 nM) for 24 h. Determination of cell viability was assessed by WST-1 kit. The results are derived from at least three independent experiments run in triplicates. Data are expressed as mean ± SD.

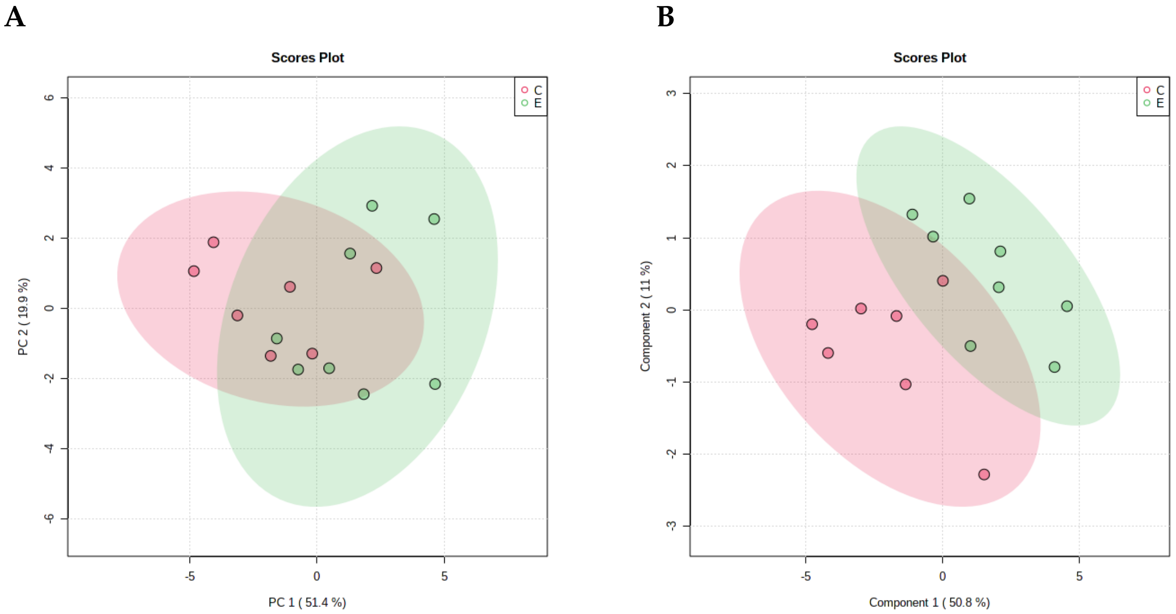

Scores plots for the first two components of PCA model (**A**) and PLS-DA model (**B**) built from spectra of plasma samples. The results of leave-one-out validation for PLS-DA model with four components: accuracy = 0.87, R2 = 0.91, Q2 = 0.51.
Symbols: (●) Control group; (●) Empagliflozin treated group.

A B

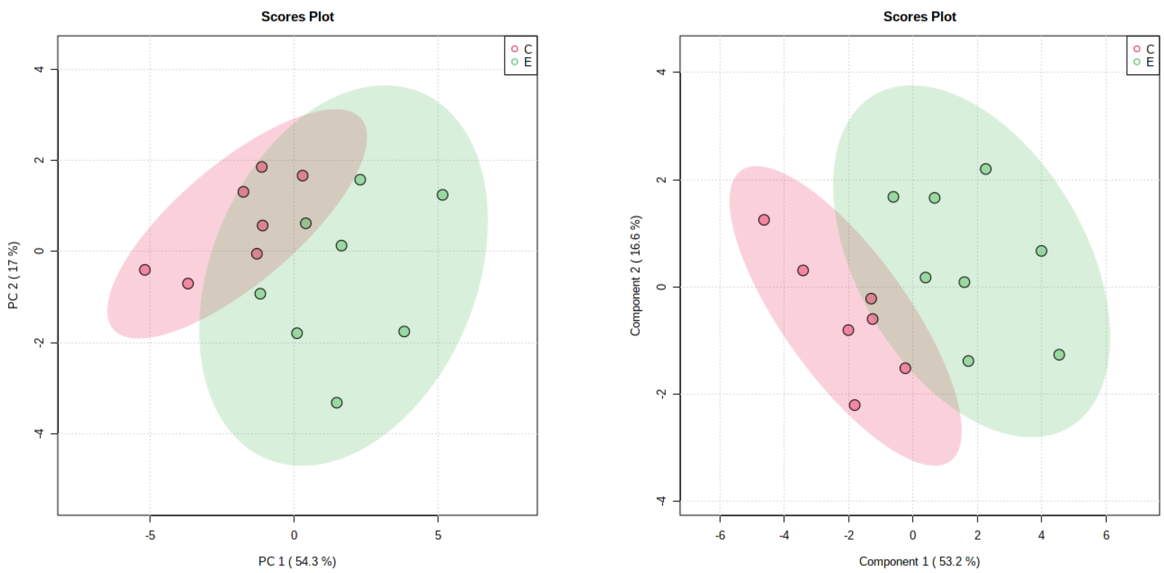

Figure S3. Scores plots for the first two components of PCA model (**A**) and PLS-DA model (**B**) built from spectra of polar liver extracts. The results of leave-one-out validation for PLS-DA model with three components: accuracy = 0.80, R2 = 0.89, Q2 = 0.41.

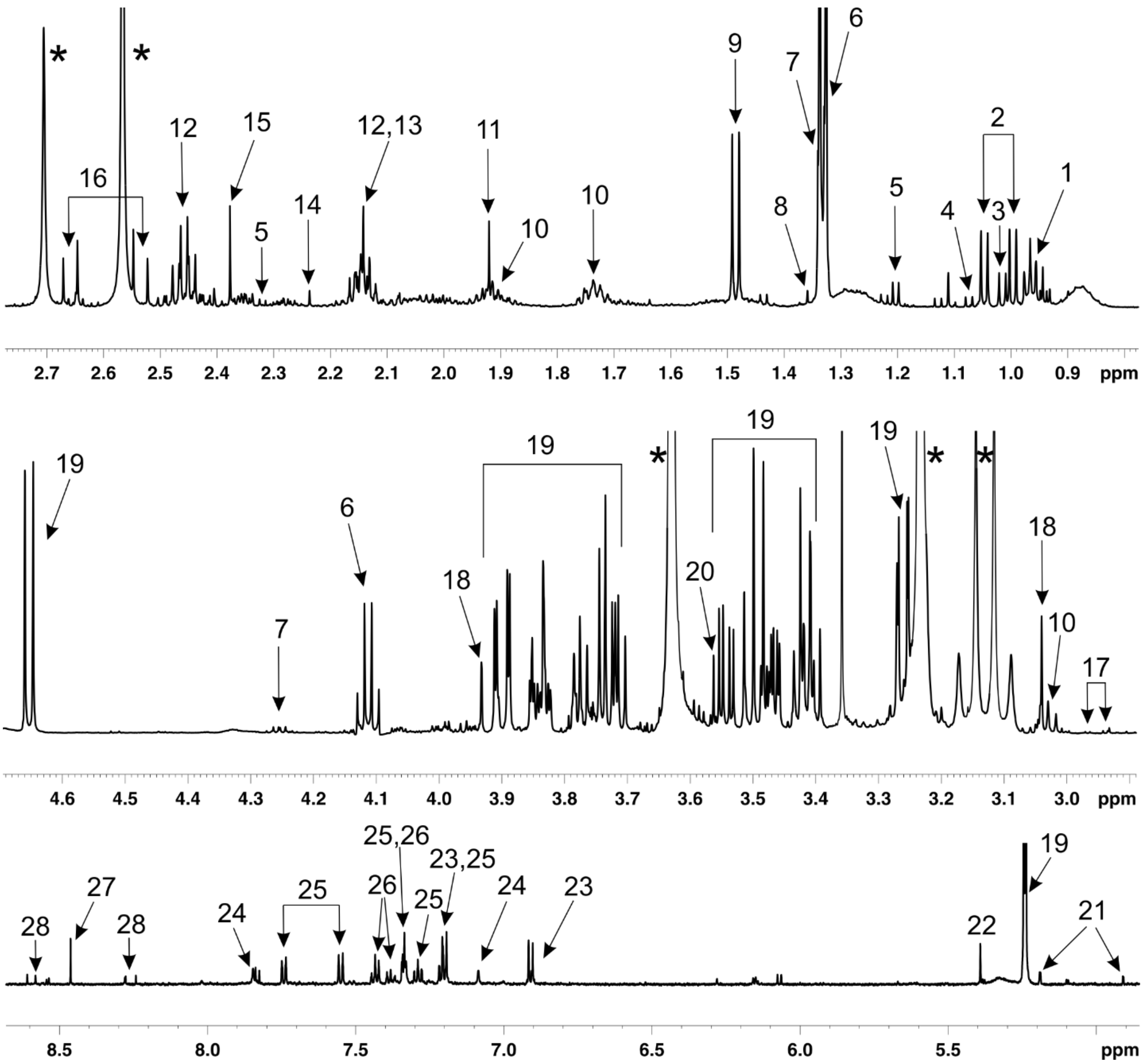

Figure S4. Representative ^{1}H NMR spectrum of plasma of hHTG rat. 1: leucine; 2: valine; 3: isoleucine; 4: isobutyrate; 5: β-hydroxybutyrate; 6: lactate; 7: threonine; 8: α-hydroxyisobutyrate; 9: alanine; 10: lysine; 11: acetate; 12: glutamine; 13: methionine; 14: acetone; 15: pyruvate; 16: citrate; 17: asparagine; 18: creatine; 19: glucose; 20: glycine; 21: mannose; 22: allantoin; 23: tyrosine; 24: histamine; 25: tryptophan; 26: phenylalanine; 27: formate; 28: ATP/ADP/AMP; *: EDTA.

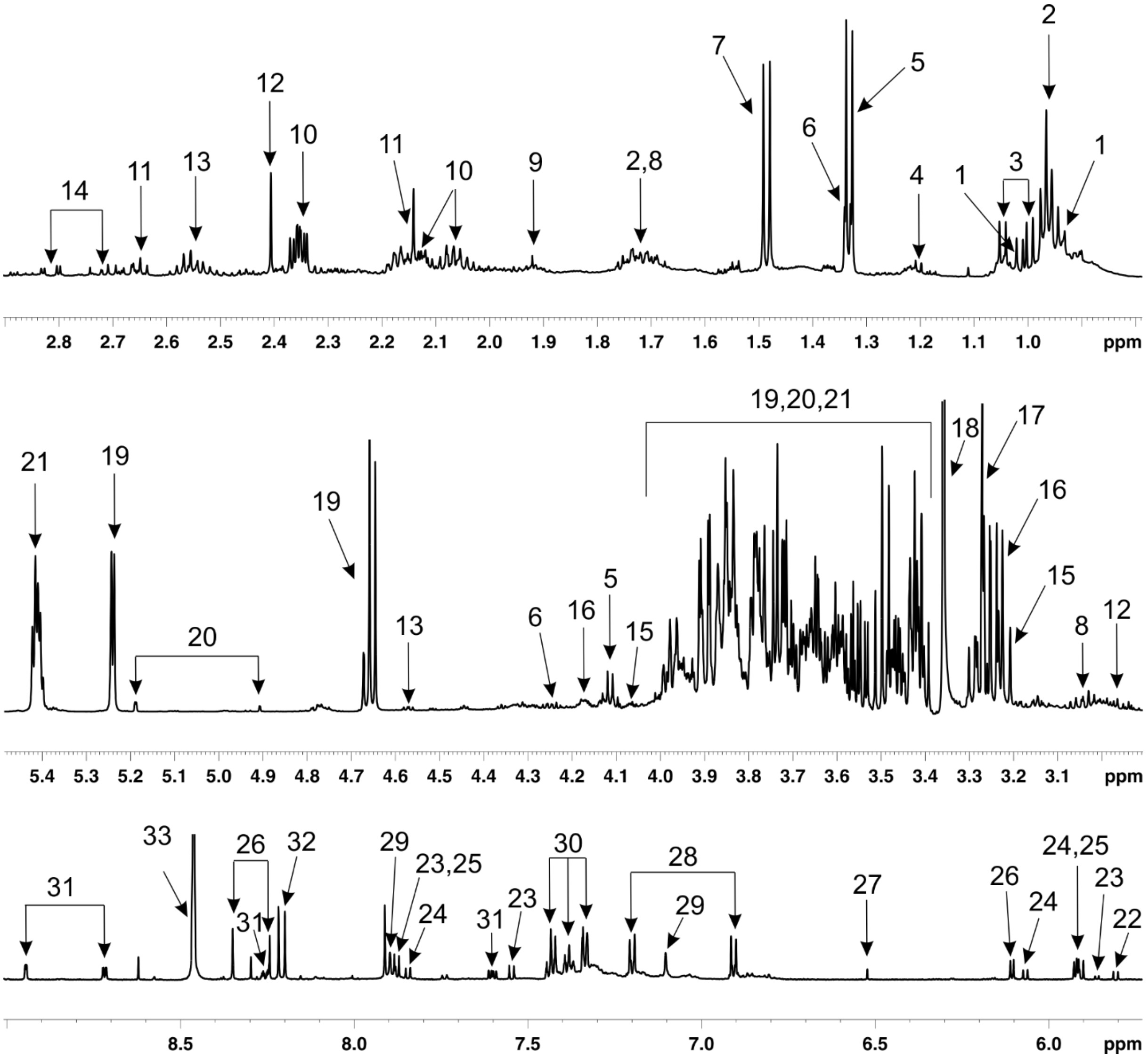

Figure S5. Representative ^{1}H NMR spectrum of polar liver extract of hHTG rat. 1: isoleucine; 2: leucine; 3: valine; 4: β-hydroxybutyrate; 5: lactate; 6: threonine; 7: alanine; 8: lysine; 9: acetate; 10: glutamate; 11: methionine; 12: succinate; 13: glutathione; 14: aspartate; 15: choline; 16: o-phosphocholine; 17: betaine; 18: methanol; 19: glucose; 20: mannose; 21: glycogen; 22: uracil; 23: xanthosine; 24: cytidine; 25: uridine; 26: inosine; 27: fumarate; 28: tyrosine; 29: histamine; 30: phenylalanine; 31: niacinamide; 32: hypoxanthine; 33: formate.

Supporting Tables

Table S1. Primer references of assessed genes for TaqMan® Gene Expression Assays.

Gene	Assay ID	Protein
Acaca	Rn00573474_m1	Acetyl-CoA carboxylase alpha
Agl	Rn01521753_m1	Amylo-alpha-1, 6-glucosidase, 4-alpha-glucanotransferase
Atgl	Rn01479969_m1	Patatin-like phospholipase domain containing 2, adipose triglyceride
Ccr2	Rn01637698_s1	Chemokine (C-C motif) receptor 2
Cd14	Rn00572656_g1	Cd14 molecule; monocyte differentiation antigen CD14
Cd36	Rn00580728_m1	Cd36 molecule; fatty acid translocase
Cd68	Rn01495634_g1	Cd68 molecule
Cidea	Rn04181355_m1	Cell death-inducing DFFA-like effector a
Cpt1a	Rn00580702_m1	Carnitine palmitoyl transferase 1A
Cpt1b	Rn00682395_m1	Carnitine palmitoyl transferase 1B
Dio2	Rn00581867_m1	Iodothyronine deiodinase 2
Fasn	Rn00569117_m1	Fatty acid synthase
Foxo1	Rn01494868_m1	Forkhead box O1
G6pc	Rn00689876_m1	Glucose-6-phosphatase
Gpam	Rn00568620_m1	Glycerol-3-phosphate acyltransferase, mitochondrial
Hmox1	Rn00561387_m1	Heme oxygenase 1
Hsl	Rn00689222_m1	Lipase E, hormone sensitive type
Ikk2	Rn00584379_m1	Inhibitor of nuclear factor kappa B kinase subunit beta
Il10	Rn01483988_g1	Interleukin 10
Il6	Rn01410330_m1	Interleukin 6
Insr	Rn00690703_m1	Insulin receptor
Kim-1	Rn00597703_m1	Havcr-1; Hepatitis A virus cellular receptor 1
Lpl	Rn00561482_m1	Lipoprotein lipase
Mcp-1	Rn00580555_m1	Chemokine (C-C motif) ligand 2
Ngal	Rn00590612_m1	Lipocalin 2
Nrf2	Rn00582415_m1	Nuclear factor, erythroid 2-like 2
$p16^{Ink4a}$	RN00580664_m1	Cyclin-dependent kinase inhibitor 2A
$p21^{waf1}$	RN00589996_m1	Cyclin-dependent kinase inhibitor 1A
Pck1	Rn01529014_m1	Phosphoenolpyruvate carboxykinase 1
Pgc-1α	Rn00580241_m1	PPARγ coactivator 1 alpha
Pgc-1β	Rn00598552_m1	PPARγ coactivator 1 beta
Pparγ	Rn00440945_m1	PPARγ; peroxisome proliferator-activated receptor gamma
Ppp1ca	Rn00580546_m1	PP1alpha; protein phosphatase 1 catalytic subunit alpha
Ppp2ca	Rn01460084_g1	PP2A-alpha; protein phosphatase 2 catalytic subunit alpha
Prkaa1	Rn00665045_m1	Protein kinase AMP-activated catalytic subunit alpha 1
Prkab2	Rn00575219_m1	Protein kinase AMP-activated non-catalytic subunit beta 2
Prkag3	Rn01400849_m1	Protein kinase AMP-activated non-catalytic subunit gamma 3
Ptpn1	Rn01423685_m1	Protein tyrosine phosphatase, non-receptor type 1
Rela	Rn01502266_m1	Nuclear factor NF-kappa-B p65 subunit
Sirt1	Rn01428096_m1	Sirtuin 1
Sglt1	Rn01640634_m1	Solute carrier family 5 member 1
Sglt2	Rn00574917_m1	Solute carrier family 5 member 2
Socs3	Rn00585674_s1	Suppressor of cytokine signalling 3
Tgfβ1	Rn00572010_m1	TGF-β; transforming growth factor, beta 1
Timp2	Rn00573232_m1	TIMP metallopeptidase inhibitor 2
Tnf	Rn99999017_m1	TNF-α; tumor necrosis factor

Table S2. Primer references of control gene for TaqMan® Gene Expression Assays.

Gene	Assay ID	Protein
18s	Hs99999901_s1	Eukaryotic 18S rRNA
B2m	Rn00560865_m1	Beta-2-microglobulin
Gapdh	Rn01775763_g1	Glyceraldehyde-3-phosphate dehydrogenase
Gusb	Rn00566655_m1	Glucuronidase, beta

Table S3. Primer sequences for mRNA quantification in cell lines.

Gene	GenBank	Species	Forward (5'−3')	Reverse (5'−3')
p21^{Waf1}	NM_007669.5	Mus musculus	TCGCTGTCTTGCACTCTGGTGT	GTGGGCACTTCAGGGTTTTCTC
p21^{WAF1}	NM_000389.5	Homo sapiens	TCACTGTCTTGTACCCTTGTGC	GGCGTTTGGAGTGGTAGAAA
B2m	NM_009735.3	Mus musculus	GCCTGTATGCTATCCAGAAAACC	CGGGTGGAACTGTGTTACG
B2M	NM_004048.4	Homo sapiens	GGCTATCCAGCGTACTCCAAAG	CTTCAATGTCGGATGGATGAAACC
